# Mechanical ventilation and long-term neurocognitive impairment after acute respiratory distress syndrome

**DOI:** 10.1186/s13054-020-2736-7

**Published:** 2020-01-31

**Authors:** Giovanni Giordano, Francesco Pugliese, Federico Bilotta

**Affiliations:** Department of Anaesthesia and Intensive Care, University La Sapienza, Rome, Italy

Dear Editor,

We read with great attention and interest the paper by Sasannejad et al. on long-term cognitive impairment after acute respiratory distress syndrome (ARDS) [1].

In this comprehensive review, the authors report data that widely range from epidemiology and pathophysiology to possible therapies, from hypoxemia and delirium to mechanical ventilation (MV).

The long-term neuropsychological sequelae and impaired health status in ARDS survivors were first described by Hopkins et al. in 1999 who reported that “[...]at 1 year after ARDS 30% of patients still exhibited generalized cognitive decline” and “78% patients had at least one of the following: impaired memory, attention, concentration and/or decreased mental processing speed” [2].

MV could be a pivotal mechanism that increases the risk for long-term cognitive impairment, but the complex effects and pathophysiology of MV on neuroinflammation—as well as related neurocognitive adverse outcomes—are not fully understood [3].

That appropriate MV plays a role in neurocognitive outcome is proven by the study by Beitler et al. [4]. In their retrospective propensity-adjusted analysis of MV-treated patients after out-of-hospital cardiac arrest (OHCA) resuscitation, the authors reported that “lower tidal volume [...] is independently associated with favorable neurocognitive outcome (primary endpoint), more ventilator-free days, and more shock-free days”.

Given this background, we wonder if we are facing the necessity to extend the information process to patients and their relatives—also for the proposal of informed consent—about the benefits and potential cognitive disfunction associated to MV.

## Authors’ response

Cina Sasannejad, E. Wesley Ely and Shouri Lahiri

Dear Editor,

We greatly appreciate Giordano et al.’s interest in our paper and are grateful for their insights. Given the preponderance of published data at basic and clinical levels of analysis that associate mechanical ventilation with cognitive deterioration, we agree with Giordano et al. that this risk should be disclosed as informed consent is obtained to implement mechanical ventilation.

While we also agree that tidal volume minimization appears to be neuroprotective in patients with anoxic brain injury after cardiac arrest [4], more precise risk stratification for mechanical ventilation in the general population may necessitate consideration of additional factors, including the extent and type of any pre-existing neurodegenerative substrate or duration of mechanical ventilation. For instance, although recently published data show accelerated Alzheimer’s disease neuropathology in mechanically ventilated mice, as evidenced by increased levels of the amyloid-β peptide and cognition-relevant neuroinflammation, there were differential effects of mechanical ventilation on blood-brain barrier permeability between mice with and without pre-existing neurodegenerative pathology [5]. These findings illustrate the potential complexity of the relationship between mechanical ventilation exposure and neurodegenerative processes, which may be modulated by factors related to mechanical ventilation, such as tidal volume and duration of mechanical ventilation, as well as the patient’s individual cerebral substrate. Further, the neurocognitive effects of various sedative drugs, which frequently accompany low-tidal volume mechanical ventilation, remain an active area of research [6].

The heterogeneity of critical illness and its underlying mechanisms may ultimately require a personalized approach to assessing the risks and benefits of mechanical ventilation for individual patients in the context of their overall disease state and potential for recovery. Indeed, neuroprotective approaches to mechanical ventilation may improve outcomes after cardiac arrest or brain injury [4, 7]; however, further research is still needed to clarify how neuroprotective approaches to mechanical ventilation inform the balance of risks and benefits of mechanical ventilation in other disease conditions and clinical contexts.

## Data Availability

Not applicable.
